# Aesthetic and Functional Outcomes of Combined Use of Extended Spreader Graft and Septal Extension Graft

**DOI:** 10.3390/life15040546

**Published:** 2025-03-26

**Authors:** Yung Jin Jeon, Tae-Hun Lee, Yeon-Hee Joo, Sang-Wook Kim

**Affiliations:** 1 Department of Otorhinolaryngology, Gyeongsang National University Hospital, Jinju 52727, Republic of Korea; blazeth87@gmail.com (T.-H.L.); astroflower77@hanmail.net (S.-W.K.); 2 Institute of Medical Sciences, Gyeongsang National University, Jinju 52727, Republic of Korea; silverwing002@hanmail.net; 3 Department of Otorhinolaryngology, Gyeongsang National University Changwon Hospital, Changwon 51472, Republic of Korea

**Keywords:** deviated nose, caudal septal deviation, septorhinoplasty, nasal reconstruction, extended spreader graft, septal extension graft

## Abstract

Deviation of the cartilaginous midvault and caudal nasal septum can cause both aesthetic misalignment and functional impairment of the nasal valves. This study explores the technical considerations and outcomes of combining extended spreader graft and septal extension graft in septorhinoplasty to correct these deviations. A retrospective analysis of 24 patients who underwent primary septorhinoplasty between January 2022 and July 2023, performed by a single surgeon, was conducted with a mean follow-up of 11.28 months. Unilateral or bilateral extended spreader grafts and septal extension grafts were placed on the concave side of the deviation. Clinical charts, surgical records, standardized photographs, and acoustic rhinometry data were analyzed for objective and subjective outcomes. Among 24 patients (21 males, 3 females; mean age 35.2 ± 15.0 years), successful correction of C-shaped nasal deviation was achieved with no significant complications observed in the short-term follow-up (*p* < 0.0001). Functional improvements were observed in nasal volume and minimum cross-sectional area (*p* < 0.0001). Most patients reported high satisfaction with both functional and aesthetic outcomes. Compared to traditional septorhinoplasty techniques, this combined approach offers a structured method to address both cosmetic and functional concerns. These findings suggest that the combined use of extended spreader grafts and septal extension grafts offers a promising approach for addressing both cosmetic and functional concerns in septorhinoplasty.

## 1. Introduction

Nasal deviation, including cartilaginous midvault and caudal septal deviation, presents both aesthetic and functional challenges in nasal surgery. Among these factors, deviation of the cartilaginous midvault stands as a key concern in deviated noses, being closely linked to the twist in the cephalocaudal L-strut of the nasal septum [1]. The correction of the cartilaginous dorsum deviation poses a significant and intricate challenge for rhinoplasty surgeons. Midvault deviation significantly contributes to narrowing of the internal nasal valve, inducing nasal obstruction. Surgical correction of midvault deviation holds paramount importance in reinstating optimal nasal function and achieving desirable aesthetic outcomes.

On the other hand, caudal septal deviation emerges as a predominant etiological element, often leading to enduring nasal obstruction even following septoplasty and showing a common association with cases requiring revision septoplasty [2,3]. Notably, beyond its role in causing an asymmetrical division of nasal volume, caudal septal deviation intricately correlates with external nasal valve stenosis [4]. Furthermore, from an aesthetic vantage point, the caudal septal deviation can instigate nasal tip deformities by disrupting the central tip support mechanism [5,6]. Various techniques have been described to straighten and stabilize the caudal nasal septum. Traditional approaches, including spreader grafts and septal extension grafts, have demonstrated variable success rates, with some studies reporting challenges such as cartilage warping or insufficient structural reinforcement in severe deviation cases [7,8]. Given these limitations, our study explores the combination of extended spreader grafts and septal extension grafts to optimize both functional and aesthetic outcomes. This combined approach is designed to provide enhanced structural stability while addressing the challenges associated with traditional methods.

We first present a comprehensive investigation into the effectiveness of combining extended spreader grafts with septal extension grafts, considering both aesthetic and functional aspects. This combined approach was selected to address the limitations of individual techniques by providing enhanced structural support and improved midvault stabilization. The objective of this study was to provide a thorough understanding of the correction of caudal septal deviation and centering of the cartilaginous nasal dorsum.

## 2. Materials and Methods

### 2.1. Patients and Study Design

In this study, we conducted a retrospective review of clinical data from consecutive patients diagnosed with caudal septal deviation and deviated nose who underwent primary septorhinoplasty performed by a single surgeon (YJJ) between January 2022 and July 2023. All patients were of Asian descent. Patients with a minimum follow-up of six months and adequate standardized photographs were selected for analysis. We reviewed medical records, including surgical graphic records and preoperative and postoperative standardized photographs of the face. Patients with prior nasal surgery or incomplete postoperative measurements were excluded. The Institutional Review Board of Gyeongsang National University Hospital approved the study (IRB No. 2023-04-017), and all patients provided written consent for the use of their photographs.

### 2.2. Surgical Techniques

#### 2.2.1. Approach

All enrolled patients underwent primary septorhinoplasty under general anesthesia using an open or endonasal approach. Preoperatively, we planned to use the extended spreader graft paired with a septal extension graft technique for patients with C-shaped deviation of the cartilaginous nasal dorsum and caudal septal deviation who desired both nasal obstruction improvement and aesthetic enhancement to correct midvault deviation.

#### 2.2.2. Septum Management

The septum was approached anteriorly after dome division, considering the potential need for future tip modification. We corrected any significant septal deviation and harvested the posterior portion of the septal cartilage, leaving a cartilaginous L-strut that was at least 1 cm wide for graft purposes. When necessary for septal deviation correction, we removed the perpendicular plate of the ethmoid bone and the vomer bone.

#### 2.2.3. Autologous Cartilage Harvest and Grafting

Autologous cartilage was used in all cases. If there was insufficient septal cartilage, we harvested costal cartilage from the sixth and seventh ribs. To straighten the remaining caudal cartilaginous septum, we carved the septal extension graft as a single piece in a quadrangular shape with the following dimensions: ≥1.5 mm (thickness), ≥1 cm (width), and ≥1 cm (length). The graft was inserted on the concave side in a plane perpendicular to the axis of the curvature. In most cases, a unilateral septal extension graft using septal cartilage was sufficient. If such a graft was inadequate to straighten the caudal septum, we sutured one more septal extension graft using 5-0 absorbable polydioxanone on the contralateral side.

**Figure 1:**
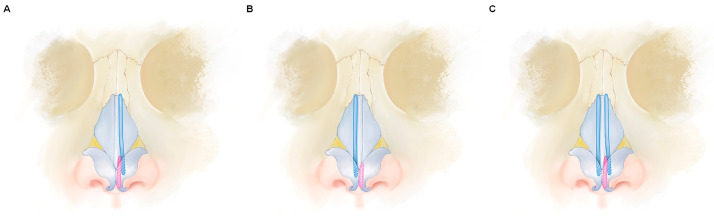
Illustration of an extended spreader graft (shown in blue) used to correct a deviated cartilaginous midvault, combined with a septal extension graft (shown in red) at the concave side of caudal septal deviation. Insertion of a unilateral extended spreader graft occurs at the concave side of the C-shaped midvault deviation, either in the same position as (**A**) or opposite to (**B**) the septal extension graft. If further correction is required, bilateral extended spreader grafts are added (**C**).

The extended spreader graft was carved and fabricated as one piece with the following dimensions: ≥1.5 mm (thickness), ≥4 mm (width), and ≥2.5 cm (length). After carving, it was inserted on the concave side of the C-shaped deviation of the dorsal portion (Figure 1A,B). We used quilting mattress sutures with 5-0 absorbable PDS and Vicryl sutures (Ethicon, Cincinnati, OH, USA) to secure the framework to the native L-strut and then straightened and stabilized it in a midline position. If an additional correction was needed for the deviated midvault, we added the extended spreader graft on the contralateral side of the pre-existing graft (Figure 1C).

#### 2.2.4. Tip Modification and Dorsum Augmentation

After placing the extended spreader grafts and septal extension grafts, we performed adjunctive procedures on the tip and radix using septal cartilage grafts to achieve a harmonious profile according to the desired tip projection and dorsal height. We employed tip onlay grafting or alar cephalic-resection techniques for tip modification. Dorsal augmentation was performed with a glued diced cartilage graft [9]. For dorsal camouflage, we used homologous fascia lata or acellular allogenic dermal matrix (MegaDerm™; L&C BIO, Seongnam-si, Republic of Korea). In cases of nasal bone deviation, conventional lateral or medial nasal bone osteotomies were performed.

### 2.3. Measurements

#### 2.3.1. Anthropometric Measurements

**Figure 2:**
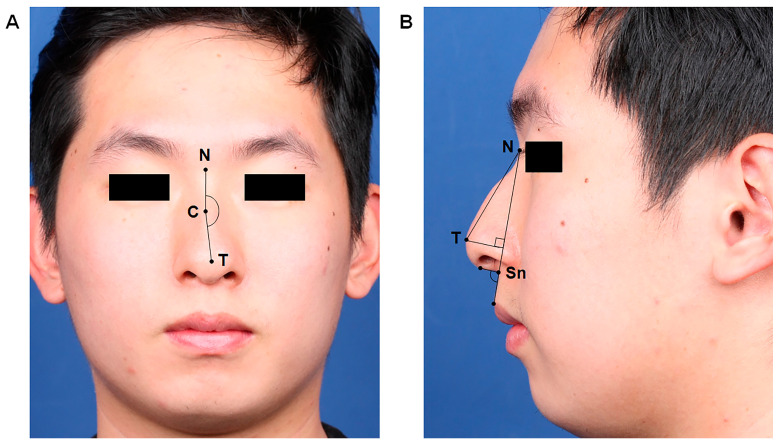
Anthropometric measurements for dorsal deviation angle (**A**), facial angle, and tip projection (**B**). (**A**) The dorsal deviation angle was defined as the angle between the line drawn from the nasion (N) to the most prominent point of the convexity of the C-shaped deviation (C) and the line drawn from the most prominent point of the convexity (C) to the nasal tip (T) on the frontal view. (**B**) The nasolabial angle represents the angle formed by the line tangent to the columella intersected by the subnasale-to-labial limb line. The tip-projection length between the nasal tip and the subnasale point was measured. To determine the tip projection, we measured Goode’s ratio using a line drawn from the alar–facial groove to the nasal tip (T) on the lateral view to calculate the distance from the nasion (N) to the nasal tip (T). Abbreviations: N, nasion; C, most prominent point of the convexity of the C-shaped deviation; T, nasal tip; Sn, subnasale.

For anthropometric analysis, we used frontal and lateral views of standardized preoperative and postoperative photographs for each patient. We noted changes in the dorsal deviation angle, nasolabial angle (NLA), and tip-projection length (Figure 2). To ensure objective evaluations, two nasal surgeons (YHJ and THL), who were blinded to the clinical information, analyzed the anthropometric factors. Each anthropometric measurement was performed using Adobe Photoshop 2020 (Adobe Systems Inc., San Jose, CA, USA).

#### 2.3.2. Acoustic Rhinometry

We used impulse acoustic rhinometry (GMI Ltd., Cambridge, UK) for the objective measurements of nasal obstruction, which were recorded by a single trained technician throughout the study. Recordings were acquired according to a published user manual. As directed, the patient was prepared before testing in a quiet, stable environment for 15–20 min. The experienced technician, who was blinded to patient variables, analyzed three satisfactory recordings from each nasal cavity. The values for the narrow-side nasal cavity were averaged. We obtained measurements for the minimal cross-sectional area (MCA, cm^2^) and nasal cavity volume (cm^3^).

#### 2.3.3. Questionnaires for Functional and Aesthetic Outcomes

In addition, the Nasal Obstruction Symptom Evaluation (NOSE) scale was adopted for functional outcome evaluation [10]. An aesthetic surgical outcome evaluation was completed through patient surveys according to postoperative aesthetic surgical outcomes were assessed using the 10-Item Standardized Cosmesis and Health Nasal Outcomes Survey (SCHNOS) [11,12].

### 2.4. Statistical Analysis

All statistical analyses were performed using SPSS 25.0 (IBM Corporation, Armonk, NY, USA), and the data are presented as mean (±standard deviation). Mann–Whitney U testing was performed to compare the variables between the two independent groups. Wilcoxon signed-rank testing was performed to compare the preoperative and postoperative anthropometric factors. All tests were two-sided, with *p* < 0.05 being considered statistically significant.

## 3. Results

### 3.1. Demographics and Preoperative Patient Characteristics

A total of 24 patients was enrolled in this study, including 21 men and 3 women. The mean age of these patients was 35.21 ± 15.02 years, with an age range of 19–81 years. The average duration of follow-up was 11.28 ± 4.71 months, ranging from 6.17 to 20.27 months. Preoperative findings related to the direction and location of septal deviation and midvault deviation are summarized in Table 1. Among the included patients, 8 had a moderate degree and 16 had a severe degree of caudal septal deviation. Other preoperative coexisting deformities included saddle nose (n = 2) and hump nose (n = 13).

### 3.2. Surgical Details

Regarding the surgical approach, 21 patients were treated using an external approach, while three were treated with an endonasal approach (details provided in Table 1). Autologous rib cartilage grafts were harvested for 13 patients, while, for the remaining patients, sufficient septal cartilage was available. Six patients requiring bilateral extended spreader grafts had a greater degree of dorsal deviation compared to patients who received unilateral extended spreader grafts (*p* < 0.0001). However, there was no significant difference in functional factors such as preoperative values of nasal cavity volume, MCA, or NOSE score (Table 2).

Adjunctive procedures included dorsal camouflage using diced cartilage wrapped in homologous fascia lata or MegaDerm™ for dorsal augmentation in 14 patients, while seven patients required one layer of homologous fascial lata or MegaDerm™ for dorsal camouflage. Tip plasty, which involved techniques such as tip onlay graft, alar cephalic resection, or a combination, was performed in 21 patients to achieve the desired tip projection. Additionally, seven patients underwent lateral or medial osteotomies to correct nasal bone deviations.

### 3.3. Changes in Anthropometric Factors and Aesthetic Assessment

**Figure 3:**
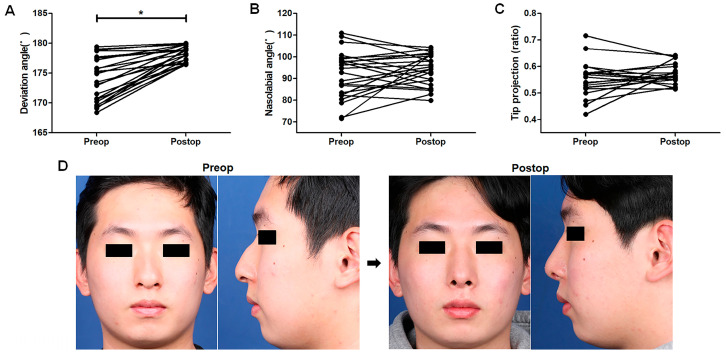
Improvements in aesthetic outcomes following surgery. Changes in the anthropometric factors of the dorsal deviation angle (**A**), nasolabial angle (**B**), and Goode’s ratio for tip projection (**C**) before and after surgery. Exemplary patient photographs showcase successful midvault deviation correction and improved nasal contour (**D**) six months after surgery. * *p*-values when comparing the values before and after the surgical procedure.

The dorsal deviation angle, NLA, and Goode’s ratio for tip projection were measured and compared before and after surgery in all enrolled patients. Based on the findings, the average deviation angle improved from 173.90° ± 3.66° to 178.30° ± 1.29° (*p* < 0.0001, Figure 3A). The 4.40° correction in the dorsal deviation angle represents a visible and functional improvement in nasal alignment, reducing asymmetry. Although the change in NLA (from 91.76° ± 10.79° to 94.17° ± 7.29°, *p* = 0.370, Figure 3B) was not significant, it moved toward the ideal Asian angle [13]. Goode’s ratio for tip projection increased slightly from 0.55 ± 0.013 to 0.57 ± 0.008 (*p* = 0.199, Figure 3C). The general aesthetic contours of the nose were also improved after surgery (Figure 3D), and the postoperative aesthetic domain from the SCHNOS revealed satisfactory results (2.79 ± 0.41 points) postoperatively. Based on these findings, significant changes were observed in the dorsal deviation angle, indicating that midvault deviation correction was successful, and the general aesthetic contour of the nose was improved after surgery.

### 3.4. Changes in Acoustic Rhinometry Components and Functional Improvement

**Figure 4:**
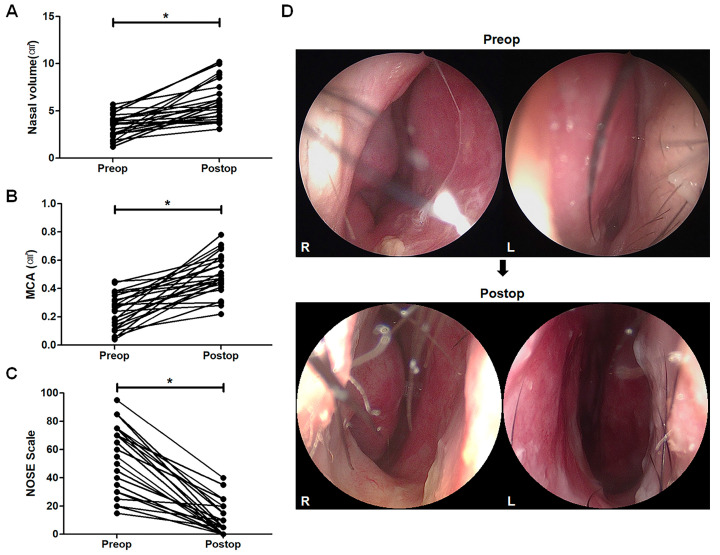
Improvements in functional outcomes following surgery. Acoustic rhinometry outcomes exhibit changes in nasal volume (**A**) and the minimal cross-sectional area (MCA, (**B**)), as well as improvements in the NOSE score (**C**). Representative endoscopic images depict the successful correction of caudal septal deviation (**D**) six months after surgery. * *p*-values when comparing the values before and after the surgical procedure.

The components of AR showed significant improvement from preoperative to postoperative assessment. Postoperatively, the mean nasal volume was increased from 3.41 ± 0.26 cm^3^ to 5.88 ± 0.48 cm^3^ (*p* < 0.0001, Figure 4A), while the MCA increased from 0.24 ± 0.12 cm^2^ to 0.50 ± 0.14 cm^2^ (*p* < 0.0001, Figure 4B). The NOSE score indicated improved breathing postoperatively, with scores decreasing from 53.13 ± 4.96 points to 10.42 ± 2.39 points in all patients (*p* < 0.0001, Figure 4C). A 72% increase in nasal volume and a 108% increase in MCA reflect substantial airflow enhancement, which correlates with the significant improvement in NOSE scores. These findings suggest that patients not only experience improved nasal patency but also a meaningful reduction in obstruction symptoms. The representative nasal endoscopic examination revealed successful correction of the caudal nasal septum alignment six months after the surgery (Figure 4D). No residual nasal obstruction was observed during final follow-up visits. No major complications or recurrence occurred.

## 4. Discussion

Deviated cartilaginous midvault and caudal septal deviation are frequently encountered issues in septorhinoplasty procedures [1]. Our study presents the first comprehensive analysis of clinical objective outcomes and patient-reported assessments related to centering of the cartilaginous nasal dorsum and the correction of caudal septal deviation by using autologous cartilage. The combination of extended spreader and septal extension grafts effectively corrects midvault and caudal septal deviations. This technique allowed for the correction of both the external and internal nasal valves, which play crucial roles in nasal function.

Nasal septal deviation typically contributes to external deviation of the nasal midvault [14,15,16]. However, understanding the complexities of a deviated nose involves consideration of multiple factors. Acquired injuries often exert external forces on the nasal septum, leading to the deformation of nasal bones and upper lateral cartilage as well as their connection to septal bones like the ethmoid bone, vomer, and maxillary crest [16]. Intrinsic forces may also arise due to imperfect septal cartilage growth or tissue structural changes caused by trauma, further contributing to cartilage deviation, and the combination of these extrinsic and intrinsic forces results in a deviated nose. The most common deformities, such as C-shaped (left-side concavity) or reverse C-shaped (right-side concavity) deformity [14], can be caused by a tilted bony pyramid with a correspondingly tilted or bent cartilaginous vault, or by a straight bony pyramid a tilted or bent cartilaginous vault [15]. Correcting a deviated nasal septum is paramount in single-stage septorhinoplasty for managing a deviated nose, although additional procedures, such as separation and reconstruction using various grafts or sutures of unilateral or bilateral upper lateral cartilage and correction of bony pyramid through osteotomy, may also be necessary for comprehensive management.

Spreader grafts, initially conceptualized by Sheen et al. [17], have significantly advanced the correction of dorsal deviations, strengthening the middle nasal vault and preventing postoperative nasal valve collapse. By broadening the angle of the internal nasal valve, spreader grafts also enhance respiratory airflow. Though various techniques described in the literature are performed to modify the cartilaginous portion of the dorsal septum and straighten the nose [18,19], their efficacy demands extensive follow-up and validation using large sample sizes due to potential issues like cartilage warping and structural weakening. Jang et al. previously demonstrated the effectiveness of combining caudal septal extension grafts with unilateral extended spreader grafts for centering the cartilaginous dorsum [20]. Our study findings align with previous research, indicating that a unilateral spreader graft on the convex side of midvault deviation is often sufficient for centering a deviated nose [20,21]. However, in severe cases, bilateral spreader grafts offer enduring correction by stabilizing the dorsal septum and counteracting deviations caused by cartilage memory.

The present study underscores the pivotal role of septal correction in addressing nasal deformities, particularly in patients presenting with caudal septal deviation, with or without high dorsal deviation. Surgical techniques encompassing realignment, weakening, and subsequent reconstruction aim to preserve cartilaginous tissue while establishing a straight foundation for the columella and cartilaginous dorsum [22]. This study highlights the use of septal extension grafts to correct nasal caudal septum deviations, enhancing tip support and controlling tip shape, projection, and rotation [23,24]. However, the present study notes challenge specific to Asian patients, such as thick nasal skin, a bulbous tip shape, and inadequate tip support [25,26,27]. necessitating additional procedures like tip onlay grafts or alar cephalic resection. Our results revealed promising aesthetic changes, including a trend toward the ideal Asian nasolabial angle and tip projection, suggesting favorable aesthetic changes, although these changes were not significant. The aesthetic enhancements observed in our study were supported by the subjective aesthetic outcome survey, with patients reporting high levels of satisfaction.

Preoperative imaging plays a crucial role in the accurate assessment and planning of septorhinoplasty procedures. Various imaging modalities, including plain X-rays, sinus sonography, and computed tomography (CT) scans, provide valuable insights into structural abnormalities of the nasal framework. CT scans, in particular, offer high-resolution imaging, enabling precise evaluation of bony and cartilaginous deviations. Ultrasonography has also been explored as a non-invasive diagnostic tool for assessing nasal and paranasal sinus conditions [28]. Given the importance of these imaging techniques, incorporating standardized imaging protocols in the preoperative evaluation may enhance diagnostic accuracy and improve surgical planning. Further research incorporating imaging modalities as part of routine preoperative assessment may help refine patient selection and optimize surgical strategies.

Both deviated cartilaginous midvault and caudal septal deviation affect upper lateral cartilage collapse on the narrow side and a narrow nasal valve area, resulting in symptoms of nasal obstruction [4]. Our study demonstrated substantial functional improvements through quantitative measurements and patient-reported outcomes. Although no major complications occurred, minor complications were observed. Two patients (8.3%) who had severe facial asymmetry experienced mild asymmetry, though it did not significantly affect nasal function or aesthetic outcomes. As this is a retrospective study, selection bias may affect the results, potentially limiting their generalizability. Moreover, the small sample size (24 patients: 21 males, 3 females) reduces statistical power, restricting broader applicability. To strengthen the evidence, future prospective, multi-center studies with larger cohorts should be considered. Additionally, our average follow-up duration of 11.28 months, while providing valuable short-term data, may be insufficient for assessing long-term graft stability. Future studies should extend follow-up periods to at least two years. Furthermore, excluding patients with previous nasal surgeries limits the applicability of our findings to revision cases, which are common in real-world surgical practice. Future research should include revision cases to assess the effectiveness of this technique in more complex nasal deformities. While this study provides valuable insights into the effectiveness of the technique for the Asian population, we plan to extend the research to other ethnic groups with diverse nasal structures in future studies to improve the broader applicability of the results. Additionally, our study utilized acoustic rhinometry as an objective airflow assessment method. Future studies could incorporate additional techniques such as rhinomanometry or computational fluid dynamics (CFD) for a more detailed analysis of airflow resistance and functional outcomes.

## 5. Conclusions

Correction of deviated cartilaginous midvault and caudal septal deviation poses a multifaceted challenge in septorhinoplasty. The strategic integration of extended spreader grafts and septal extension grafts demonstrates promising functional and aesthetic improvements with high patient satisfaction rates. However, this study is limited by its retrospective design, small sample size, and lack of a control group, which may affect the generalizability of the findings. Future research should include randomized controlled trials and longer follow-up studies to further assess the long-term stability of grafts and refine surgical techniques. Despite these limitations, our findings support the use of extended spreader grafts and septal extension grafts as a valuable approach for addressing midvault and caudal septal deviations in septorhinoplasty.

## Figures and Tables

**Table 1:** Clinical characteristics and surgical details of patients (N = 24).

Characteristics	Number (%) or Mean ± SD
Age (years)	35.21 ± 15.02
Gender distribution Male Female	21 (87.5) 3 (12.5)
Allergic rhinitis	17 (70.8)
Direction of midvault deviation Right Left	11 (46) 13 (54)
Direction of caudal septal deviation Right Left	6 (25) 18 (75)
Location of septal deviation Caudal Caudal and Dorsal	7 (29.2) 17 (70.8)
Degree of septal deviation Moderate Severe	8 (33.3) 16 (66.7)
Other coexisting deformities Saddle nose Hump nose	2 (8.3) 13 (54.2)
**Surgical details**	**Number (%)**
Approach External Endonasal	21 (87.5) 3 (12.5)
Type of reconstruction graft Rib cartilage Septal cartilage	13 (54.2) 11 (45.8)
Direction of extended spreader graft Right Left Bilateral	7 (29.1) 11 (45.8) 6 (25.0)
Direction of septal extension graft Right Left Bilateral	16 (66.7) 7 (29.2) 1 (4.1)
Other procedures Dorsal onlay graft Glued diced cartilage Homologous fascia lata or MegaDerm™ for camouflage	21 (87.5) 14 (58.3) 7 (29.2)
Tip plasty Tip onlay graft Alar cephalic resection	21 (87.5) 19 (79.2) 5 (20.8)
Osteotomy Lateral Medical and Lateral	7 (29.2) 5 (20.8) 2 (8.3)
**Mean duration of follow-up (months)**	11.28 ± 4.71

**Table 2:** Comparison between unilateral and bilateral extended spreader graft placement.

		Extended Spreader Graft Placement		*p*-Vaule
		Unilateral (N = 18)	Bilateral (N = 6)	
Functional factor	NV	3.60 ± 1.25	2.85 ± 1.02	0.168
	MCA	0.25 ± 0.12	0.19 ± 0.13	0.305
	NOSE scale	10.44 ± 5.19	11.17 ± 4.07	0.733
Aesthetic factor	Dorsal deviation angle	175.17 ± 3.21	170.10 ± 1.83	<0.001 *

NV, nasal volume; MCA, minimal cross-sectional area; NOSE, Nasal Obstruction Symptom Evaluation. * *p*-values < 0.05 indicate statistical significance.

## Data Availability

The data supporting the findings of this study are not publicly available due to privacy and ethical restrictions. However, they can be made available from the corresponding author upon reasonable request.

## List of Abbreviations

NLA, nasolabial angle; MCA, minimal cross-sectional area; NOSE, Nasal Obstruction Symptom Evaluation; SCHNOS, Standardized Cosmesis and Health Nasal Outcomes Survey; CT, computed tomography; CFD, computational fluid dynamics.
